# Characteristics of control group participants who increased their physical activity in a cluster-randomized lifestyle intervention trial

**DOI:** 10.1186/1471-2458-11-27

**Published:** 2011-01-11

**Authors:** Lauren A Waters, Marina M Reeves, Brianna S Fjeldsoe, Elizabeth G Eakin

**Affiliations:** 1 The University of Queensland, School of Population Health, Cancer Prevention Research Centre, Brisbane, Queensland, Australia

## Abstract

**Background:**

Meaningful improvement in physical activity among control group participants in lifestyle intervention trials is not an uncommon finding, and may be partly explained by participant characteristics. This study investigated which baseline demographic, health and behavioural characteristics were predictive of successful improvement in physical activity in usual care group participants recruited into a telephone-delivered physical activity and diet intervention trial, and descriptively compared these characteristics with those that were predictive of improvement among intervention group participants.

**Methods:**

Data come from the Logan Healthy Living Program, a primary care-based, cluster-randomized controlled trial of a physical activity and diet intervention. Multivariable logistic regression models examined variables predictive of an improvement of at least 60 minutes per week of moderate-to-vigorous intensity physical activity among usual care (n = 166) and intervention group (n = 175) participants.

**Results:**

Baseline variables predictive of a meaningful change in physical activity were different for the usual care and intervention groups. Being retired and completing secondary school (but no further education) were predictive of physical activity improvement for usual care group participants, whereas only baseline level of physical activity was predictive of improvement for intervention group participants. Higher body mass index and being unmarried may also be predictors of physical activity improvement for usual care participants.

**Conclusion:**

This is the first study to examine differences in predictors of physical activity improvement between intervention group and control group participants enrolled in a physical activity intervention trial. While further empirical research is necessary to confirm findings, results suggest that participants with certain socio-demographic characteristics may respond favourably to minimal intensity interventions akin to the treatment delivered to participants in a usual care group. In future physical activity intervention trials, it may be possible to screen participants for baseline characteristics in order to target minimal-intensity interventions to those most likely to benefit. (Australian Clinical Trials Registry, http://www.anzctr.org.au/default.aspx, ACTRN012607000195459)

## Background

Physical activity is implicated in the prevention and management of numerous chronic diseases, including a reduced risk of cardiovascular disease, cancer, diabetes, musculoskeletal disorders, anxiety and depression [1,2]. However, national surveillance surveys conducted in Australia and other developed countries reveal that only 40-60% of the population participate in levels of physical activity sufficient to derive health benefits [3-6]. Consequently, research into the development and dissemination of effective, broad reaching physical activity interventions is an important public health priority.

A rapid increase in the number of reports of physical activity intervention studies in the peer-reviewed literature [7,8] has occurred since the release of the Surgeon General's report on physical activity and health in 1996 [1]. However, despite an increased research focus, the results of several meta-analyses suggest that overall intervention effects in many physical activity intervention trials across population groups, intervention settings and delivery modalities are modest, with mean effect sizes ranging from 0.28 to 0.50 [9-13]. Furthermore, the long-term effectiveness of physical activity interventions is uncertain, with few interventions reporting on the maintenance of outcomes following the end of an intervention [9].

A potential contributor to modest effect sizes in physical activity intervention trials is the concurrent increase in physical activity in both control and intervention group participants, which could attenuate intervention effects. Our recent systematic review found that in almost 30% of physical activity intervention trials in primary care-based settings, a significant and clinically meaningful improvement of at least 60 minutes per week of moderate-to-vigorous physical activity was observed in the control group (unpublished data). A number of potential explanations for control group improvements have been posited, including: the Hawthorne effect (where participants improve in the experimental variable being tested due to awareness of being observed) [14-16]; social desirability bias (the propensity to report behaviour that is compatible with social norms) [17-19]; regression to the mean (a problem associated with intra-participant variation and measurement error, which may occur in trials using pre- and post-intervention measurements, particularly when behavioural screening is employed to select an inactive sample) [20-22]; the effects of measurement (when measurement is sufficient to produce a change in behaviour in the absence of a formal intervention) [16,23]; or, the recruitment of a highly motivated volunteer sample.

It is also possible that control group improvements could be attributable to the minimal level of intervention often delivered to control group participants in physical activity intervention trials (e.g., brief advice, standardised print materials). As brief interventions that result in increases in physical activity are likely to be highly cost-effective, determining whether there is an association between individual characteristics and meaningful physical activity improvement for participants allocated to the control, or less intensive treatment arm of randomised trials, is an important endeavour and could have important implications for public health practice.

The Logan Healthy Living Program, a 12-month telephone and print-delivered physical activity and diet intervention targeting patients with type 2 diabetes or hypertension, is an example of a methodologically rigorous intervention trial in which a significant control group improvement in physical activity occurred [24]. In this trial, significant intervention effects were observed for all dietary outcomes. However, there was no significant intervention effect for moderate-to-vigorous intensity physical activity, owing to concurrent statistically and clinically meaningful improvements in both the usual care (UC) and telephone counselling (TC) groups. At 12-months, the TC group reported a mean improvement of 71.2 (SE 14.3) minutes of moderate-to-vigorous physical activity per week while the UC group improved by 84.5 (SE 14.9) minutes per week [25], and these improvements were maintained in both groups six-months later [26].

To our knowledge, no previous study has investigated whether certain individual characteristics are predictive of physical activity improvements specifically for control group participants. Predictors of control group improvement may be different to predictors of improvement in intervention group participants, as the mechanisms underlying control group change are poorly understood. The purpose of the current study is to identify baseline demographic, health and behavioural characteristics that were associated with a meaningful physical activity improvement among control group participants in the Logan Healthy Living Program. We further compare these to the predictors of improvement in the intervention group.

## Methods

This paper uses data from a cluster-randomized controlled trial, the Logan Healthy Living Program. A detailed description of the methods for this trial has been published elsewhere [24]. Ethics approval was granted by The University of Queensland, Human Research Ethics Committee. Data were collected from February 2005 to November 2007, with the present analysis conducted in November 2009.

### Participant Recruitment

Participants were recruited from primary care practices in a socio-economically disadvantaged community bordering Brisbane (the capital city of the state of Queensland), Australia. Ten primary care practices consented to participate (28% of those contacted and deemed eligible) [24] and were randomly assigned by simple random allocation to either the telephone counselling intervention (TC) or usual care (UC) condition. Within practices, patients with type 2 diabetes or hypertension, who were 30 years or older and had a listed telephone number, were identified via searches of electronic medical records. Patient lists were screened by general practitioners (GPs) and patients with contraindications to participation in an unsupervised physical activity and diet intervention were excluded. Participants were not excluded from participation based on screening for baseline levels of physical activity or dietary intake.

Patients identified by their GP as being eligible to participate were sent a recruitment letter (with a reply paid decline form if they did not want to be contacted about the study), and subsequently received a phone call from study staff, during which eligibility was confirmed and consent was solicited. Of 2,172 patients identified from electronic medical records, 1,319 (61%) were sent a letter of invitation from their GP, 847 (64% of those posted a letter) were successfully contacted by phone, and 598 (71% of those contacted by phone) were deemed eligible to participate. In total, 434 patients (73% of those deemed eligible and 20% of original sample identified from electronic medical records) consented to participate - 228 participants received the telephone counselling (TC) intervention and 206 received the usual care (UC) condition.

### Telephone Counselling Intervention

Briefly, the TC group participants received up to 18 telephone calls over 12-months. The frequency of telephone calls was highest in the first four months of the trial (weekly for the first month, then fortnightly) and tapered to monthly calls for the remaining eight months. TC participants were also mailed a pedometer and a detailed workbook with information on physical activity and healthy eating. The TC intervention was underpinned by Social Cognitive Theory [27] and the Social Ecological Model [28-30] and employed motivational interviewing techniques [31].

### Usual Care Condition

As this was a cluster-randomised controlled trial, with randomisation conducted at the level of the GP practice, all eligible patients from UC practices were invited to participate in a 'Healthy Living Program'. Participants in the UC group were not aware that there was a more intensive treatment arm. To limit attrition over the duration of the study, participants in the UC group were posted a project newsletter at baseline, 4-, 8- and 12-months. The newsletter contained general health tips, although not on physical activity and diet, and off-the-shelf brochures addressing a variety of health topics related to diabetes and hypertension, including weight loss, healthy food choices, sugar and alcohol intake, physical activity, heart disease, blood pressure, foot care, managing stress and depression and sexual health. Following each assessment, which coincided with the baseline, 4- and 12-month mailings, participants in the UC group also received a 1-page 'thank-you' letter with brief feedback on their assessment results. Feedback was a single sentence that listed (in bullet point format) the health behaviours for which they were not meeting recommended guidelines.

### Measures and Data Collection

Data were collected at baseline, 4-, 12- and 18-months by computer-assisted telephone interviewers who were blind to study condition allocation. Only baseline and 12-month data are included in this analysis.

### Dependent Variable

The primary dependent variable for this investigation was meaningful change in minutes of moderate-to-vigorous intensity physical activity undertaken per week. A meaningful change was defined as an increase of 60 minutes or more per week based on evidence demonstrating that one hour per week of physical activity is associated with a reduction in risk for all cause mortality, cardiovascular disease and indicators of metabolic disease [2,32-34].

Physical activity was assessed using the Active Australia Survey, a 6-item self-report survey assessing total time and number of sessions per week spent in vigorous and moderate physical activities, and walking over the last seven days. This survey has been reported to have acceptable criterion validity compared to accelerometer measures [35,36] and acceptable agreement with the U.S. Behavioural Risk Factor Surveillance Survey (BRFSS), and the International Physical Activity Questionnaire (IPAQ) [37]. Test-retest reliability coefficients for the Active Australia Survey are comparable to those reported for the U.S. BRFSS, and the IPAQ [38]. Total minutes of physical activity per week were calculated as the sum of walking, moderate and 2 × vigorous minutes per week, with maximum minutes per week being truncated at 1680 [39]. Change in weekly minutes of physical activity was calculated by subtracting baseline total minutes from 12-month total minutes. The change variable was then recoded into a dichotomous variable in order to categorise participants into those who had and had not increased their physical activity by 60 minutes or more per week between baseline and 12-months.

### Predictor Variables

For this analysis, the following self-reported baseline demographic, health-related and behavioural predictor variables were considered:

#### Demographic predictors

Age, gender, ethnicity, education level, employment status, weekly household income, and marital status.

#### Health-related predictors

Body mass index (BMI) category (healthy: 18.5 - 24.9 kg/m^2^, overweight: 25-29.9 kg/m^2^, obese: ≥30 kg/m^2 ^[40]) and the total number of chronic conditions (summed from a self-reported checklist including: diabetes, cardiovascular disease, hypertension, hypercholesterolemia, stroke, arthritis, lung disease, cancer, depression and anxiety) and categorised as 1-2, 3-4 or ≥5 chronic conditions.

#### Behavioural predictors

Baseline smoking status (current smoker or not) and physical activity (reported as hours of moderate-to-vigorous physical activity per week).

### Statistical Methods

Data analysis was conducted using SPSS for Windows (version 17). We compared the baseline characteristics of participants in the UC and TC groups, and the characteristics of participants who completed the 12-month physical activity assessment (completers) versus those who dropped out. Statistically significant, or clinically meaningful (≥ 10%) differences are reported. The main analysis for this study uses data from completers, none of whom had missing data for predictor variables, (n = 166 UC; n = 175 TC). There was some evidence of selective dropout, therefore a sensitivity analysis using all randomised participants (n = 206 UC; n = 228 TC) and assuming no change from baseline for those who dropped out of the study, was conducted to examine whether results were robust to the selective drop out.

Bivariate analyses comparing participants who did and did not demonstrate a meaningful improvement in physical activity across a broad range of baseline variables were conducted using chi-square tests (categorical variables), t-tests (normally distributed continuous variables) or the Mann-Whitney test (non-normal, continuous data). Analyses were stratified by group because there were significant differences between UC and TC groups in the associations of meaningful change with some predictors (baseline physical activity, education; p for interaction <0.05). Independent associations were tested using logistic regression analyses, with results reported as odds ratios with 95% confidence intervals. In addition to age and gender, categorical variables were earmarked for inclusion in logistic regression models if there was a 10% difference between groups with and without a meaningful improvement, or if results were significant at the p < 0.20 level [41]. Continuous variables were considered for inclusion in logistic regression models if results were significant at the p < 0.20 level [41]. A backwards elimination process was then undertaken to remove the unnecessary variables from the models. The least significant variables were removed sequentially until all remaining variables in the models were significant at the p < 0.20 level and there was no statistically significant difference between the full models and the reduced models. Models showed no evidence of multicollinearity. We did not correct analyses for clustering as there was no evidence of clustering in physical activity outcomes in the Logan Healthy Living Program trial [25]. Significance was set at the conventional level (p < 0.05; two tailed).

## Results

### Baseline characteristics

The study had a high rate of participant retention with approximately 80% of enrolled participants completing follow up assessments. Within the UC group, participants who were excluded from the main analyses (n = 40) differed from the included sample (n = 166) with respect to income and BMI, with more excluded UC participants reporting 'don't know' or refusing to answer questions about household income (30.0% [n = 12] of excluded vs. 13.3% [n = 22] of included, p = 0.043), and being in the obese BMI category (62.5% [n = 25] of excluded vs. 39.8% [n = 66] of included, p = 0.008). Within the TC group, participants who were excluded from the main analyses (n = 53) differed from included participants (n = 175) with respect to smoking status with more excluded TC participants being current smokers (26.4% [n = 14] of excluded vs. 8.0% [n = 14] of included, p = 0.001). These differences reinforce the need to conduct sensitivity analyses.

Overall, approximately 50% of participants who completed the study reported a meaningful improvement in moderate-to-vigorous physical activity of 60 minutes or more per week. Baseline demographic and health-related data for the 166 UC and 175 TC participants included in the primary analysis are presented in Table 1. The majority of participants were women (62%) and Caucasian (92%). The mean age of participants was 58 (±12) years; 83% were overweight or obese; and the majority reported having 3-4 chronic conditions at baseline. There were no significant differences between the TC and UC groups for age, gender, ethnicity, education, income, employment, marital status or participation in sufficient physical activity at baseline (Table 1). There were, however, significant between group differences in the number of current smokers (TC = 8%, UC = 18.1%, p = 0.007) and the number of obese participants (TC = 52.6%, UC = 39.8%, p = 0.045).

**Table 1:** Characteristics of Logan Healthy Living Program participants with complete data at baseline and follow up

Baseline Characteristic	Telephone counselling group n = 175	Usual care group n = 166
Age (years)	58.6 ± 12.0	57.7 ± 11.9
Gender (female)	65.1 (114)	59.6 (99)
Ethnicity (Caucasian)	92.0 (161)	92.8 (154)
Education Primary school	21.1 (37)	18.7 (31)
Secondary school	43.4 (76)	47.6 (79)
Technical or trade diploma	22.9 (40)	21.1 (35)
University	12.6 (22)	12.7 (21)
Employment		
Full time	29.1 (51)	31.9 (53)
Part time/casual	17.7 (31)	14.5 (24)
Retired	38.3 (67)	36.7 (61)
Home duties	6.9 (12)	10.8 (18)
Unable to work/unemployed/student	8.0 (14)	6.0 (10)
Weekly household income ($)		
200-299	11.4 (20)	9.6 (16)
300-499	20.6 (36)	20.5 (34)
500-999	21.7 (38)	19.3 (32)
1000-1499	20.6 (36)	18.1 (30)
1500+	13.7 (24)	19.3 (32)
Don't know/refused	12.0 (21)	13.3 (22)
Married or cohabiting	69.7 (122)	73.5 (122)
Current smoker*	8.0 (14)	16.9 (28)
BMI category		
Healthy	15.4 (27)	18.7 (31)
Overweight	32.0 (56)	41.6 (69)
Obese	52.6 (92)	39.8 (66)
Number of chronic health conditions		
1-2	35.4 (62)	44.6 (74)
3-4	45.7 (80)	39.8 (66)
≥5	18.9 (33)	15.7 (26)
Meeting physical activity guidelines (150 minutes of moderate-to-vigorous PA/week and ≥5 sessions)	25.1 (44)	25.9 (43)

Data are presented as % (n) or mean ± SD

*significant between groups difference

### Multivariable logistic regression analysis

#### Usual care group

At 12-months, 51% (n = 84) of UC group participants reported an increase of 60 minutes or more of moderate-to-vigorous physical activity. Based on the results of the bivariate analyses, the following variables were found to be associated with physical activity improvement and entered into the multivariable adjusted logistic regression model: BMI category, education, income, employment, and marital status, along with age and gender. A backwards stepwise elimination process resulted in age being removed from the model. After mutual adjustment for all variables included in the model, BMI category, education, marital status and employment status remained significant predictors of meaningful improvement in physical activity at 12-months for the UC group (Table 2). The odds of reporting a meaningful improvement in physical activity at 12-months increased as baseline BMI category increased. Compared to those with a healthy weight, the odds ratio for demonstrating a meaningful increase in physical activity was 5.01 (CI = 1.62, 15.47, p = 0.005) for participants who were obese at baseline and 3.68 (CI = 1.25, 10.87, p = 0.018) for people who were overweight at baseline. Participants who had a primary school education (OR = 0.28, CI = 0.10, 0.77, p = 0.014), or who had completed a trade qualification (OR = 0.24, CI = 0.09, 0.66, p = 0.006), were significantly less likely than participants with a secondary school education (but not tertiary qualifications) to have increased their physical activity at 12-months. The odds of reporting a meaningful improvement in physical activity were significantly higher for participants who were not married or cohabiting with a partner (OR = 2.61, CI = 1.03, 6.60, p = 0.042), compared to those who were married. Retired participants were more likely to increase physical activity by 60 minutes per week than participants who were employed full time (OR = 5.88, CI = 1.80, 19.21, p = 0.003).

**Table 2:** Predictors of a meaningful* increase in physical activity from baseline to 12-months (usual care group)

	n = 166	OR	95% CI	p-value
***Sex***				
Male	67	1.00		
Female	99	0.48	(0.21, 1.13)	0.093
***BMI Category***				
Healthy	31	1.00		0.018
Overweight	69	3.68	(1.25, 10.87)	0.018
Obese	66	5.01	(1.62, 15.47)	0.005
***Education***				
Secondary school	79	1.00		0.010
Primary school	31	0.28	(0.10, 0.77)	0.014
Trade or tech diploma	35	0.24	(0.09, 0.66)	0.006
University	21	0.66	(0.21, 2.04)	0.471
***Weekly household income***				
200-299	16	1.00		0.110
300-499	34	0.29	(0.07, 1.26)	0.099
500-999	32	1.33	(0.25, 7.15)	0.742
1000 - 1499	30	1.55	(0.26, 9.29)	0.632
>1500	32	1.63	(0.25, 10.72)	0.611
Don't know/refused	22	1.09	(0.21, 5.66)	0.916
***Marital status***				
Married or cohabiting	122	1.00		
Not married or cohabiting	44	2.61	(1.03, 6.60)	0.042
***Employment status***				
Full time	53	1.00		0.045
Part time/casual	24	2.07	(0.63, 6.82)	0.230
Retired	61	5.88	(1.80, 19.21)	0.003
Home duties	18	3.49	(0.87, 14.01)	0.078
Unable to work/unemployed/student	10	1.76	(0.30, 10.42)	0.532

n = number in group, OR: odds ratio, CI: confidence interval

*Meaningful increase is defined as a change of 60 minutes per week from baseline to 12-months.

#### Telephone counselling group

At 12-months, 52% (n = 91) of TC group participants reported an increase of 60 minutes or more of moderate-to-vigorous physical activity. Baseline moderate-to-vigorous physical activity in hours per week was included in the initial logistic regression model, as were age and gender. Age and gender, were subsequently removed in a backwards, stepwise elimination process. Baseline physical activity therefore remained the only significant predictor of meaningful improvement in physical activity at 12-months after adjusting for the other variables. For every additional hour of physical activity reported by participants at baseline, the odds of demonstrating a meaningful change in physical activity at 12-months were reduced by 20% (OR = 0.80, CI = 0.71, 0.91, p≤0.001).

#### Sensitivity analyses

For the TC group, carrying forward baseline values for physical activity for those with missing data had no impact on the variables found to be significant predictors of a meaningful improvement in physical activity at 12 months. However, in the sensitivity analysis for the UC group, several variables found to be predictive of a meaningful improvement in physical activity in the primary completers' model were no longer significant predictors (i.e., BMI category and marital status). For the UC group, significant predictors of improvement in physical activity at 12 months were education level and employment status.

## Discussion

This study found that certain socio-demographic characteristics (being retired and having a secondary school education) consistently predicted an increase in physical activity of 60 minutes or more per week among participants allocated to the usual care arm of a physical activity and diet intervention trial. Other socio-demographic variables (being married, and being overweight or obese) were found to be predictors of meaningful improvement in an analysis of all intervention completers, but not when participants who dropped out were included in a sensitivity analysis assuming no improvement in physical activity. In contrast, for participants in the telephone counselling intervention group, baseline physical activity was the only predictor of a meaningful improvement in physical activity at 12 months, and this association remained in both the completers and sensitivity analyses.

These findings suggest that, in the primary care setting, participants with certain socio-demographic characteristics may particularly benefit from brief-intensity physical activity interventions (akin to that provided to the UC group in this trial). It should be noted that the impact of undergoing assessments cannot be distinguished from the effects of receiving the brief intervention. However, measurement itself could be considered to be, and used as, an intervention. Measurement has been shown to impact on participants' physical activity in the intervention trial context [23]. Brief behavioural assessments have been shown to successfully reduce hazardous drinking [42], and the same may be true for physical activity.

To our knowledge, this is the first study to investigate predictors of meaningful physical activity improvement among participants randomised to the control or usual care group. Previous studies that have examined predictors of physical activity change have either used data from the intervention group only [43,44] or pooled data from intervention and control groups [45,46]. The results of the current study suggest that this latter approach may be inappropriate, given that predictors of successful physical activity improvement appear to differ for the control and intervention groups. This may be because predictors of physical activity improvement in response to an intervention are dependent on the intensity of that intervention. There is also limited evidence available on predictors of physical activity improvement for brief interventions [47,48], thus there are no directly comparable studies to support or contrast our findings for participants in the UC group in this trial.

The predictors identified for the UC group do make intuitive sense. As suggested in other studies, having less than a secondary school level of education may impair people's to ability to interpret and respond to brief health promotion messages [49], and retired participants may have more time available to consider and act upon the brief assessment feedback and standardized print materials provided [50]. The associations between baseline marital status or BMI and meaningful physical activity improvement are inconclusive; however it is possible that participants who are not married or who are overweight may respond favourably to minimal intensity interventions. Participants who are married may have greater time constraints related to home or family life, leading to an actual or perceived reduction in the amount of time available to dedicate to increasing physical activity [51], and having a higher baseline BMI has been identified as a predictor of improvement in physical activity in several studies [52]. However these findings, particularly the association between physical activity improvement and BMI, should be interpreted cautiously. UC group participants with a higher BMI were more likely to drop out of the trial; therefore it is possible that the association between baseline BMI and a meaningful improvement in physical activity may be an artefact of selective drop out, with BMI actually being a predictor of study retention.

Targeting minimally intensive interventions towards people with certain socio-demographic profiles could be a particularly cost-effective approach to improving population levels of physical activity. Stepped care models triage individuals to varying levels of care or intervention based on pre-treatment characteristics or response to treatment [53,54] and have been applied to other health behaviours, most notably, smoking cessation interventions [55]. Several interventions aimed primarily at promoting weight loss, but which have also addressed physical activity, have adopted a stepped care approach [56,57]. In such an approach, participants would be assigned to a level of intervention based on baseline demographic characteristics, and progressed to more intensive levels of intervention if they failed to make satisfactory behaviour changes in response to initial treatments. This stepped care approach could be applied to physical activity interventions, however, the effectiveness of such an approach, and the baseline socio-demographic profile on which it might be based, are areas requiring further research[53].

In contrast to the findings in the UC group, the more intensive telephone-delivered intervention evaluated in this trial was equally effective across socio-demographic characteristics. A low baseline level of physical activity was the only predictor of a meaningful improvement in physical activity for the participants in the TC group. Two previous studies that specifically evaluated predictors of physical activity change among intervention group participants also found that being less active was predictive of greater improvement [43,44]. In contrast to our findings, these two studies also reported that some socio-demographic characteristics (namely age, gender, ethnicity and level of education) were also predictive of physical activity improvement. It makes intuitive and statistical sense that participants with lower levels of physical activity would report greater improvements in physical activity in response to an intervention because they have a greater capacity for change. It is also likely that a more intensive intervention is necessary to assist inactive people to make meaningful increases to their physical activity; and this may explain why baseline physical activity was a predictor for participants in the TC group, but not for those in the UC group.

This study has several limitations. This was a secondary, exploratory analysis that lacked the experimental manipulation necessary to infer causation. As psychosocial and cognitive variables were not measured in this trial, we were not able to assess the capacity of these to predict control group improvements. While assessing these characteristics would require a more intensive screening process, some research suggests that they are potentially more useful in predicting changes in physical activity than socio-demographic variables [48]. A broader range of predictive variables may be necessary to more accurately predict successful behaviour change. However, the socio-demographic, health-related and behavioural variables included in this study might be more feasible to employ as screening tools to target minimal-intensity interventions than psychosocial and cognitive variables. Physical activity was collected via self-report measures. In comparison to objective measures, self-reported physical activity may be over-reported due to recall inaccuracy or bias [58], or under-reported due to the constraints of questions asked (e.g. only reporting physical activity that occurs in bouts greater than 10 minutes duration) [58]. In the context of this study, measurement error could lead to misclassification of participants who did and did not demonstrate a meaningful change in physical activity. However, any biases in measurement should operate equally for the control and intervention groups. Finally, results from this study may be specific to the study sample and intervention. Future work on control group predictors is needed to determine the extent to which these results are generalisable. Interventions of greater or lesser intensities, with different control group protocols (e.g., no control group contact beyond measurement), and using different definitions of successful change may find different variables to be predictive of physical activity improvements.

## Conclusions

This study examined whether certain characteristics were predictive of meaningful physical activity improvements among participants assigned to the usual care arm of a telephone-delivered, primary care-based intervention trial. The identification of socio-demographic variables that were predictive of successful physical activity improvement for usual care group participants suggests that it would be useful to examine a stepped care approach for physical activity intervention trials. In such an approach, screening for baseline socio-demographic characteristics could be used to target minimal intensity interventions to those most likely to benefit. Such methods have not been implemented as part of an intervention in which physical activity is the primary outcome. More work is needed to identify the characteristics most likely to be predictive of improvement in response to minimal levels of intervention, and such analyses could easily be conducted in the context of existing and future trials.

## Competing interests

The authors declare that they have no competing interests.

## Authors' contributions

LW, MR, BF and EE were involved in the conceptualisation of the study. LW carried out the analyses and drafted the manuscript. MR provided guidance and assistance in conducting the analyses, interpreting the data, and drafting of the manuscript. EE and BF also provided assistance in interpretation of the data and drafting of the manuscript. All authors read and approved the final manuscript.

## Pre-publication history

The pre-publication history for this paper can be accessed here:

http://www.biomedcentral.com/1471-2458/11/27/prepub
